# Effect of Eccentric Exercise on Metabolic Health in Diabetes and Obesity

**DOI:** 10.1186/s40798-023-00596-2

**Published:** 2023-09-29

**Authors:** Gergő Szűcs, Márton Pipicz, Márton Richárd Szabó, Tamás Csont, László Török, Csaba Csonka

**Affiliations:** 1 Metabolic Diseases and Cell Signaling (MEDICS) Research Group, Department of Biochemistry, Albert Szent-Györgyi Medical School, University of Szeged, Dóm tér 9, Szeged, 6720 Hungary; 2 Centre of Excellence for Interdisciplinary Research, Development and Innovation of the University of Szeged, 6720 Szeged, Hungary; 3 Department of Traumatology, Albert Szent-Györgyi Medical School, University of Szeged, Semmelweis utca 6, Szeged, 6720 Hungary; 4 Department of Sports Medicine, Albert Szent-Györgyi Medical School, University of Szeged, Tisza Lajos krt. 107, Szeged, 6720 Hungary

**Keywords:** Eccentric training, Lengthening contraction, Exercise, Downhill, Prediabetes, Insulin resistance, Impaired glucose tolerance, Diabetic, Obesity, Overweight, Metabolism

## Abstract

There is a growing body of evidence showing the importance of physical activity against civilization-induced metabolic diseases, including type 2 diabetes (T2DM) and obesity. Eccentric contraction, when skeletal muscles generate force by lengthening, is a unique type of skeletal muscle activity. Eccentric contraction may lead to better power production characteristics of the muscle because eccentric contraction requires less energy and can result in higher tension. Therefore, it is an ideal tool in the rehabilitation program of patients. However, the complex metabolic effect (i.e., fat mass reduction, increased lipid oxidation, improvement in blood lipid profile, and increased insulin sensitivity) of the eccentric contraction alone has scarcely been investigated. This paper aims to review the current literature to provide information on whether eccentric contraction can influence metabolic health and body composition in T2DM or obesity. We also discussed the potential role of myokines in mediating the effects of eccentric exercise. A better understanding of the mechanism of eccentric training and particularly their participation in the regulation of metabolic diseases may widen their possible therapeutic use and, thereby, may support the fight against the leading global risks for mortality in the world.

## Key Points

Lack of physical exercise is a global public health problem.

Eccentric exercise is beneficial for patients with civilization-induced chronic metabolic disorders, i.e., overweight, obesity, and type 2 diabetes mellitus

Eccentric exercise might play a role in the prevention of T2DM and obesity diseases.

## Introduction

Diabetes (and prediabetes) is a chronic disease characterized by elevated levels of blood glucose accompanied by disturbed metabolism of fats and proteins. In T2DM, blood glucose rises because of the inability of the cells to effectively use the insulin that is being produced (insulin resistance), and over time, the production of insulin progressively decreases [1, 2]. The risk factors for T2DM are well-known. Although the genetic component is substantial, the majority of the cases occur in the presence of risk factors including age, overweight and obesity, and physical inactivity [3]. Obesity (and overweight) is one of the biggest public health challenges currently. It is defined as excessive fat accumulation due to an imbalance in energy intake and energy use [4]. Obesity is often associated with numerous comorbidities, such as metabolic disease, cardiovascular diseases, T2DM, obstructive sleep apnea, certain types of cancer, and osteoarthritis [5].

Physical activity represents the first-line prevention and treatment of chronic metabolic disorders, i.e., obesity and T2DM [6–11]. It has been suggested that exercise as a medical intervention should be prescribed in terms of its dose, i.e., mode, intensity, frequency, and duration [9, 12]. As a medical intervention, the prescription for exercise should also be specifically based on the individual's capabilities and needs [9]. Compared to concentric exercise, eccentric exercises can be performed with reduced perceived effort because of the lower metabolic demand at the same workload [13]. Comprehensive reviews are available [14–17] for detailed information on eccentric exercise and the difference between concentric and eccentric exercises.

### Eccentric Contraction and its Practical Use

Eccentric muscle contraction, whereby the muscle is actively lengthened under an external load (sometimes referred to as negative work of the muscle), rarely occurs alone during natural movements. It can be observed during "braking" movements (e.g., going down a slope or stairs, or controlled lowering of weights). It displays several molecular and neural characteristics that distinguish eccentric contractions from isometric and concentric contractions [14]. One crucial difference between them is the lower oxygen and energy requirement. Here, instead of the ATP-dependent decoupling of actin–myosin cross-bridges, actomyosin bonds are decomposed primarily because of external energy [14, 18–21]. Another feature of the eccentric contraction is that 1.2–1.8 times higher tension can be achieved when compared to isometric or concentric contraction, mainly because of the increasing resistance of the elastic elements and the increase in the level of muscle activation because of the stretching reflex, which results in the formation of new cross-bridge connections [14, 19–21].

Therefore, a unique feature of eccentric exercise is that untrained subjects become stiff and sore the day after eccentric exercising because of damage to muscle fibers (i.e., in the membrane of the myofibrils, sarcoplasmic reticulum, and mitochondria) resulting in delayed onset of muscle soreness [19, 21–23]. However, the second period of exercise, a week after the first, produces much less damage as a result of the adaptation process. The ability of the muscle to rapidly adapt following the damage induced by the eccentric exercise raises the possibility of clinical applications of mild eccentric exercises, such as protecting a muscle against more major injuries or metabolic diseases [24].

Accumulating evidence shows that eccentric exercise alone can significantly increase muscle strength and other biomechanical advantages in healthy individuals [25–27]. Eccentric training is usually inserted into various training programs to maximize muscle size, strength, and power and to increase muscle cross-sectional area, force output, and fiber shortening velocities, all of which have the potential to improve power production characteristics [28]. Physical activity, including eccentric exercise, has beneficial effects on health-related parameters like lean mass gains, fat mass reduction, increased lipid oxidation, improvement of blood lipid profile, and increased insulin sensitivity in healthy individuals [21].

Performing eccentric exercises is a safe, feasible, and efficacious supplement (mainly due to its lower metabolic cost) in various rehabilitation populations against muscle atrophy, weakness, sarcopenia, and osteoporosis [13, 29]. Eccentric exercises are now incorporated into various rehabilitation interventions in the affected patients [20, 30]. For exercise intolerance and many types of sports injuries, experimental evidence suggests that interventions involving eccentric exercise are demonstrably superior in regard to conventional concentric interventions [29]. The most popular fields of rehabilitation where eccentric exercise is used are the treatment of different tendinopathies, i.e., rotator cuff tendinopathy [31, 32], lateral elbow [33], Achilles [34–36], patellar [37, 38], or hamstring tendinopathy [39]. Besides musculoskeletal rehabilitation, several studies have discussed the effect of eccentric exercise in healthy subjects and in a wide variety of clinical conditions, such as stroke [40], ischemic heart diseases [41], diabetes [42], obesity [43], or critical illnesses [44].

### Myokines: Exercise-Induced Regulatory Factors of the Metabolism

Eccentric exercise-induced musculoskeletal adaptations include muscle hypertrophy, increased cortical activity, and changes in motor unit behavior, all of which contribute to improved muscle function [45]. In the skeletal muscle system, which is the largest organ of the body, the active use of the contractile apparatus, i.e., physical work or exercise training, also leads to the release of a high amount of various humoral factors (e.g., myokines, metabolites, non-coding regulatory RNAs, and exosomes) derived from skeletal muscle to regulate a variety of cellular metabolic processes and the crosstalk between the skeletal muscle and various other tissues and organs of the body, including the adipose tissue, bone, brain (central nervous system), pancreas, liver, gastrointestinal system, heart, and even the skeletal muscle itself. Thus, the skeletal muscle can be considered an endocrine organ as well. The collective term ‘myokine’ was established by Pedersen in 2003 to be used for cytokines, which are produced and released by contracting skeletal muscles, exerting their effects in other organs of the body through endocrine signaling pathways [46–48]. The myokines release pattern is influenced by the type of muscle contraction, the training protocol, and the exercise duration, and it interferes with the subsequent systemic metabolic effects of exercise. For this reason, eccentric training might induce the synthesis and release of different myokines compared to intensity-matched concentric exercise [49–52].

In this paper, we aimed to review the impact of eccentric exercise on whole-body metabolism and metabolic health in the presence of chronic civilization diseases, e.g., T2DM and obesity. We believe that providing a recent update and systematic discussion of this topic may contribute to a better understanding of this field and may potentiate further research focusing on the possible use of eccentric training against the development and progress of metabolic civilization diseases.

## Eccentric Exercise in Metabolic Diseases

### Literature Search Strategy

**Fig. 1:**
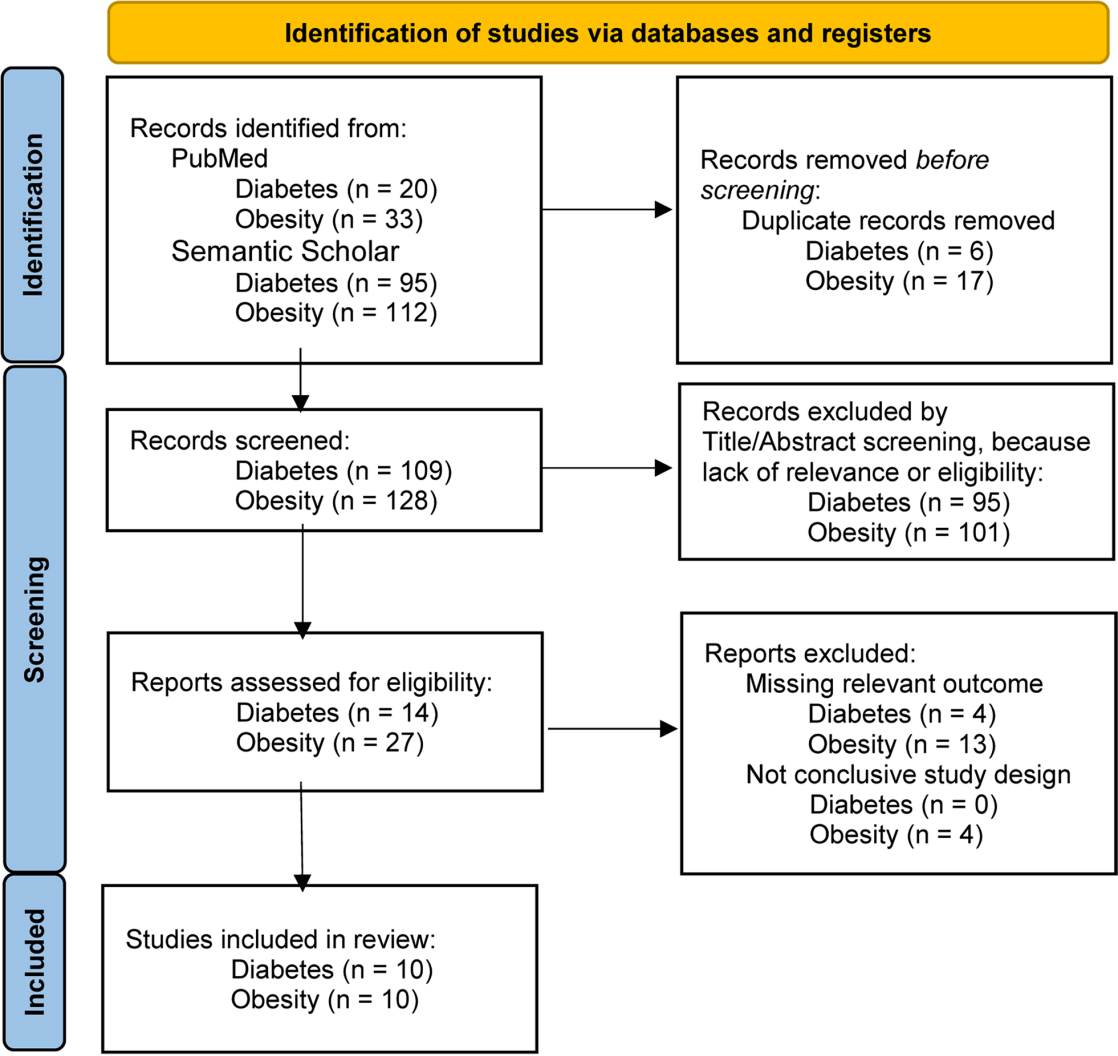
Searching strategy used to find relevant publications

The search of the literature written in English was performed in PubMed (1950–present, full-text articles) and Semantic Scholar (full-text studies, theses, and abstracts) focusing on eccentric exercise human studies with the primary outcomes related to the blood glucose and lipid homeostasis, body mass index (BMI), body composition, physical performance, and functional tests. In PubMed, the combination of the following expressions was searched using the conjunctions ‘OR’ and ‘AND’ in the Title/Abstract field that contains keyword field as well: ‘eccentric training’, ‘eccentric exercise’, ‘eccentric contraction’, ‘lengthening contraction’, ‘downhill exercise’, ‘downhill walking/running’, ‘eccentric contraction’, ‘negative work’, ‘eccentric resistance exercise’, ‘eccentric work’, ‘eccentric-only’, ‘eccentric and concentric exercise’, ‘eccentric endurance exercise’, ‘eccentric cycling’, ‘diabetes’, ‘prediabetes’, ‘prediabetic’, ‘diabetic’, ‘impaired glucose’, ‘obese’, ‘obesity’, ‘overweight’. We used the terms ‘eccentric exercise and diabetes’, and ‘eccentric exercise and obesity’ in Semantic Scholar. The last search was performed in August 2022. Figure 1 presents the PRISMA flowchart of our search strategy on “eccentric exercise on metabolic health in diabetes and obesity”.

### Eccentric Exercise in Prediabetes/Diabetes

Based on our search, 4 studies have been conducted on prediabetes [53–56] and 6 studies on T2DM [42, 57–61], which are shown in Table 1. Eccentric cycling [53, 54], eccentric stepper [42, 57], downhill walking [55, 56], downhill running [58–60], or weight-lowering resistance training alone [61] have been applied to elicit eccentric exercise. Only one study has had a bout exercise [60], others have had regular (minimum frequency 2x/week and maximum 5x/week) and long-term (between 2 and 16 weeks) exercises, usually with a progressively increased intensity (Table 1).

**Table 1:** Effects of eccentric training on prediabetic/diabetic subjects

Subjects		Eccentric intervention				Additional group	Primary outcomes	Study
Sex, n^a^	Severity of disease	Type	Duration	Intensity	Frequency of sessions, duration			
Postmenopausal female 16 (10)	Impaired glucose tolerance	Eccentric cycling	Progressive from 5’ to 20’ during the first 4 weeks, then 20–30’	Progressive RPE from 7 to 13 during the first 3 weeks, then 13	3x/ week for 12 weeks	Non-exercised control	Diff. between pre- and postexercise values (% or mean) vs controls: leg lean soft tissue mass↑, quadriceps strength↑, 6-min walking distance↑, abdominal fat ↔ , fasting plasma glucose ↔ , insulin ↔ , insulin sensitivity ↔	[53]
Males females 10 (5)	Impaired glucose tolerance	Eccentric cycling	Progressive from 5’/day to 6 × 5’/day	RPE in the range of 12–14	4-5x/ week for 2 weeks	Concentric diabetic group	vs pre-exercise value: fasting plasma glucose↑, glucose tolerance ↔ , insulin ↔ , HOMA ↔ vs concentric diabetic: insulin↑	[54]
Males 16 (8)	Impaired fasting glucose and/or glucose tolerance	Downhill walking	Mean walking time was 68’/day	RPE mean was 8.8	3x/ week for 3 weeks	Uphill walking	vs pre-exercise value: BMI ↔ , lean body mass ↔ , fasting glucose ↔ , glucose tolerance ↔ , HOMA-IR ↔ , cholesterol ↔ , triacylglycerol ↔	[55]
Males 5 (5)	Impaired fasting glucose and/or glucose tolerance	Downhill walking at low-altitude	Mean walking time was 69’/day	RPE mean was 8.9	3x/ week for 3 weeks	Same persons 1 year later; downhill walking at moderate altitude	vs pre-exercise value: blood glucose level ↔ , BMI ↔ , max heart rate ↔ (in both groups); max power output↑ (at low-altitude), antioxidant capacity↑ (at moderate altitude)	[56]
Male & female 15 (7)	Type II diabetes	Eccentric stepper + aerobic exercise	Progressive from 5’ to 20’ during the first 4 weeks, then 20–30’	Progressive from RPE 7 to 13 during the first 3 weeks, then 13	3x/ week for 16 weeks	Aerobic exercise	Compared to pre-exercise value: HbA1c↓, thigh intramuscular fat↓, thigh lean tissue↑, BMI↓, 6-min walking distance↑ Compared to aerobic exercise alone group: thigh lean tissue↑, BMI↓	[42]
Male & female 7 (7)	Type II diabetes	Eccentric stepper	Progressive from 3’ to 15’ during the first 4 weeks, than 15–30’	Progressive from RPE 7 to 13 during the first 4 weeks, than 13	3x (week1 2x)/ week for 16 weeks	No other group	Compared to pre-exercise value: insulin sensitivity↑, thigh muscle size^b^↑, muscle glucose uptake^b^↑, GLUT4↑, fasting plasma glucose ↔ , insulin ↔	[57]
- 28 (14)	Type II diabetes	Running on treadmill with slope—4°	20’ (+ additional 5–5’ warm-up and cool-down)	70% to 75% of maximum heartbeat	3x/ week for 8 weeks	Running on treadmill + 4° slope (concentric)	vs pre-exercise value: 6-min walk distance↑, blood glucose↓, HbA1c↓, lipids^c^↓ vs concentric group: 6-min walk distance↑, blood glucose↓, HbA1c↓	[58, 59]
Postmenopausal females 15 (12)	Type II diabetes	Treadmill with slope—6° before or after meals	40-min bout exercise	RPE mean was 10 before-meal and 11 after-meal groups	-, -	Sedentary; treadmill with slope + 6° before or after meals	After-meal exercise vs sedentary: postprandial insulin↓, HOMA-IR↓, osteogenic markers↑ Before-meal exercise vs sedentary: postprandial insulin ↔ , HOMA-IR ↔ , osteogenic markers ↔	[60]
Male and female 18 (9)	Type II diabetes	Cybex resistance machines; lower the weight and resist against muscle lengthening	2–3 sets of 10 repetitions; each repetition was slow controlled pace of 5-s	Progressive load from 10 to 100%	2x/ week for 12 weeks	Concentric-only; only raise or lift the weights	Compared to pre-exercise value: fasting plasma glucose ↔ , HbA1c ↔ , insulin ↔ , HOMA-2 IR ↔ , cholesterol ↔ , triacylglycerol ↔ , BMI ↔ , waist ↓, hip ↔ , waist-hip ↓, total body fat % ↓, total body lean mass ↑, 6-min walking distance↑, 5-rep chair rise time ↓, 3-m timed up-and-go time ↓ upper- and lower-body strength ↑ Compared to concentric-only group: no difference in above listed parameters	[61]

BMI: body mass index, HOMA-IR homeostatic model assessment of insulin resistance, RPE: ratings of perceived exertion on Borg scale. ↔ : unchanged, ↑, and ↓indicate an increase and a decrease, respectively. ^**a**^: number of total participants (participants in eccentric group); ^b^: sustained after one week of exercise cessation; ^c^: parameters were not specified

*Glucose homeostasis.* In prediabetic patients with impaired fasting glucose and/or impaired glucose tolerance, eccentric exercise has failed to improve blood parameters of glucose homeostasis (fasting plasma glucose, glucose tolerance, insulin, and insulin sensitivity or resistance) [53–56], independently of the sex of the patient or the type and duration of the exercise (Table 1). In one 2-week study, eccentric exercise has slightly but significantly increased fasting glucose and insulin level and HOMA index after the training period without affecting glucose tolerance [54]. In diabetes, 4 studies out of 5 have shown improvement in glucose homeostasis (e.g., HbA1c, insulin sensitivity or resistance, and blood glucose level) after regular eccentric exercise. After-meal bout exercise has also improved postprandial insulin and HOMA-IR [60] in diabetic postmenopausal women. A study that has failed to improve these parameters has somehow been different regarding the type of exercise compared to others. Participants in the ECC group were instructed to lower the weight and resist against muscle lengthening actions to elicit eccentric contractions at a guided and slow controlled pace of 5-s [61]. In diabetic patients, weight-lowering resistance training does not seem to be as effective as eccentric walking, running, or cycling (Table 1).

#### BMI and Body Composition

In the prediabetic state, regardless of the type and duration of the exercise program, neither BMI, lean body mass nor abdominal fat has been improved by eccentric training [53, 55, 56] (Table 1). Only two studies have published BMI results in T2DM. Marcus et al. have demonstrated for the first time that eccentric exercise combined with aerobic exercise is superior to aerobic exercise in diabetes regarding the BMI and thigh lean tissue parameters [42] (Table 1). The authors suggest that improvement in lean mass may beneficially influence resting metabolic rate or functional mobility [42]. Although a 12-week weight-lowering resistance training has been ineffective to influence BMI, the exercise program has decreased the total body fat and waist–hip ratio, and it has increased lean body mass [61]. Resistance exercise via negative eccentric work (RENEW) on a recumbent eccentric stepper has been shown to increase thigh muscle size in elderly diabetic patients [57]. One week after the cessation of the 16-week RENEW program, muscle size was sustained, but insulin sensitivity returned to the pretraining level.

#### Physical Performance

In prediabetes, the locomotor performance of subjects has been studied by only two research groups [53, 56] (Table 1). In a study on postmenopausal women with impaired glucose tolerance, a twelve-week knee extensor eccentric program (3 times/week) by recumbent eccentric cycling has improved quadriceps strength and 6-min walk distance compared to controls doing no exercise [53]. In a crossover study, five prediabetic men with impaired fasting glucose performed downhill walking 3 days/week for 3 weeks at a low altitude and one year later at a higher altitude [56]. The maximum power output had improved after low-altitude training, without change at moderate altitude [56]. In diabetes, eccentric exercise combined with aerobic exercise has increased 6-min walking distance; however, eccentric training has not been superior to aerobic exercise [42]. 20-min running on a −4°slope treadmill 3 times per week for 8 weeks increased the 6-min walk distance, and this improvement has been more advanced than in concentric exercise [58]. A 12-week resistance training on Cybex machine increased 6-min walk distance and upper- and lower-body strength, and it has also improved 5-repetition chair rise test and 3-m timed up-and-go test [61]. In this study, no difference has been found between eccentric and concentric trainings [61].

Taken together, the majority of the findings may suggest a favorable effect of eccentric exercise on glucose homeostasis, body composition, and locomotor performance in T2DM. Nonetheless, in prediabetic patients, eccentric exercise seems to be ineffective concerning the metabolic parameters and body composition although it may have a beneficial impact on performance.

### Eccentric Contraction and Overweight/Obesity

Ten papers were identified to investigate the effect of eccentric training in overweight or obese subjects [43, 62–70] (see Table 2). Julian et al. have compared the effects of eccentric and concentric cycling training in several studies on obese (BMI > 90th percentile) adolescents aged 12 to 16 years [43, 65, 66]. It is well known that physical exercise effectively prevents the formation of obesity and is used as an effective treatment to lose extra body weight [6, 7]. It has also been proven by Julian et al. in obese adolescents performing either concentric or eccentric training. Bodyweight and BMI significantly decreased after training, but no significant difference has been detected between eccentric and concentric groups [43, 65, 66]. Other parameters, i.e., the whole-body fat mass and leg fat mass or whole‐body lean mass and lean leg mass have been improved more effectively by eccentric training [43]. The additional positive effect of eccentric training has been shown by Thievel et al. on body mass, BMI, and fat mass [67]. It seems that eccentric training beneficially affects body weight and body composition in obese adolescents.

**Table 2:** Effects of eccentric training on overweight and obese patients

Subjects		Eccentric intervention				Additional group	Effect on primary outcomes	Study
Sex n^a^	Severity of disease	Type	Duration of sessions	Intensity	Frequency of sessions, duration			
Overweight women 22 (11)	BMI: 25–33	Eccentric exercise of knee extensors with isokinetic dynamometer Cybex Norm	55’	1 session consisting of 5 × 15 eccentric max voluntary contractions	1 session -	Lean group	serum TG↓, serum CHOL↓, LDL↓, HDL↓, resting energy expenditure↑, peak torque↓, pain-free range of movement↓, DOMS↑, CK↑	[62]
Elderly overweight and obese women 78 (14) (16)	Age: ≥ 60 yr	Isotonic exercises	50’	–	3x/ week for 12 weeks	Appropriate weight control, appropriate weight, trained overweight control, obese control	BW↓, body fat↓, fat mass↓, lean mass↑	[63]
Elderly obese women 30 (15)	Age: 60–82 yr Obesity: body fat ≥ 30%	Descending stair walking	Progressive from 2–24 repetitions, increasing the number of repetitions 2/week	110 stairs/repetitions (17 cm/stairs) (1 s/step velocity)	2x/ week for 12 weeks	Ascending stair walking group	HR↓, systolic blood pressure↓, serum glucose↓, serum insulin↓, HOMA↓, HbA1c↓, OGTT↓, serum TG↓, serum CHOL↓, LDL↓, HDL↑	[64]
Male (12) and female (12) obese adolescents 24	Age: 12–16 yr Obesity: BMI > 90 percentile	Eccentric cycling (Cyclus2 Eccentric Recumbent; RBM elektronik automation; MSE Medical, Duttlenheim, France	Phase1: 2 weeks habituation, 10–30’ Phase2: 45’ sessions (10’ wu, 30’ tr, 5’ cd) Phase3: 45’ sessions (10’ wu, 30’ tr, 5’ cd)	Phase1: if duration reached 30 min, intensity increased from VO_2_peak 20% to 50% Phase2: VO_2_peak 50% Phase3: VO_2_peak 70%	3x/ week for 12 weeks	Concentric cycling	Body weight ↓, BMI ↓, Waist circumference ↓, Hip circumference ↓, Fat mass ↓, Lean mass ↑, Whole-body fat mass (kg) ↓, Leg lean mass (kg) ↑, Leg lean mass (%) ↑, Leg fat mass (kg) ↓, Leg fat mass (%) ↓, Trunk lean mass (kg) ↓, Trunk lean mass (%) ↑, Trunk fat mass (kg) ↓, Trunk fat mass (kg) ↓, VO2peak ↑, Pmax ↑, Quadriceps strength ↑, Insulin ↓, Glucose ↓, HOMA-IR ↓	[43]
Male (16) and female (18) obese adolescents 34 (11)	Age: 12–16 yr Obesity: BMI > 90 percentile	Eccentric cycling (Cyclus2 Eccentric Recumbent; RBM elektronik automation; MSE Medical, Duttlenheim, France	Phase 1: 2 weeks habituation, 10–30’ Phase 2: 45’ sessions (10’ wu, 30’ tr, 5’ cd) Phase 3: 45’ sessions (10’ wu, 30’ tr, 5’ cd)	Phase1: if duration reached 30 min, intensity increased from VO_2_peak 20% to 50% Phase2: VO_2_peak 50% Phase3: VO_2_peak 70%	3x/ week for 12 weeks	Control and concentric cycling group	Body weight ↓, BMI ↓, whole-body lean mass (kg) ↓, whole-body lean mass (%) ↓, whole-body fat (kg) ↓, Leg lean mass (%) ↑, Leg fat mass (kg) ↑, Leg fat mass (%) ↓, isometric peak torque ↑, BMD ↑, BMC ↑	[65]
Male (11) and female (12) obese adolescents 23 (11)	Age: 12–16 yr Obesity: BMI > 90 percentile	Eccentric cycling (Cyclus2 Eccentric Recumbent; RBM elektronik automation; MSE Medical, Duttlenheim, France	Phase1: 2 weeks habituation, 10–30’ Phase2: 45’ sessions (10’ wu, 30’ tr, 5’ cd) Phase3: 45’ sessions (10’ wu, 30’ tr, 5’ cd)	Phase1: if duration reached 30 min, intensity increased from VO_2_peak 20% to 50% Phase2: VO_2_peak 50% Phase3: VO_2_peak 70%	3x/ week for 12 weeks	Concentric cycling	Body weight ↓, BMI ↓, whole-body lean mass (%) ↑, whole-body fat (%) ↓, Total VSP-A ↑, Physical well-being ↑, Energy-vitality ↑	[66]
Male (12) and female (12) obese adolescents 24	Age: 12–16 yr Obesity: BMI > 90 percentile	Eccentric cycling (Cyclus2 Eccentric Recumbent; RBM elektronik automation; MSE Medical, Duttlenheim, France	Multidisciplinary intervention Phase1: 2 weeks habituation, 10–30’ Phase2:45’ sessions (10’ wu, 30’ tr, 5’ cd) Phase3: 45’ sessions (10’ wu, 30’ tr, 5’ cd)	60 min Phase 1: if duration reached 30 min, intensity increased fromVO_2_peak 20% to 50% Phase 2: VO_2_peak 50% Phase 3: VO_2_peak 70%	1x/ week, 3x/ week for 12 weeks	Concentric cycling	BW↓, BMI↓, fat mass↓, hunger↓, desire to eat↓, preference for fat food↑, preference for sweet food↓	[67]
Men 20 (10)	Overweight	High-intensity eccentric interval cycling training	4 repetitions of 5: 2 min work-to-rest ratio training	80% of peak concentric power output	3/week for 2 weeks	High-intensity concentric interval cycling training	serum cholesterol ↓, LDL ↓, heart rate ↓, systolic blood pressure ↓	[68]
Men 22 (10)	Overweight or obese	Eccentric resistance training, squat, hip and knee flexion,	4 sets of 8 repetition per session	For the eccentric protocol, the flexion was performed during 5 s while extension 1 s	3/week for 4 weeks	Concentric resistance training	waist circumference ↓, systolic blood pressure ↓, rate pressure product ↓	[69]
Male or female	Overweight	Eccentric resistance training with dynamic resistance training machines	2–3 sets of 10–12 repetitions	75% of one repetition maximum	4/week for 12 weeks	Concentric resistance training	lean body mass ↑, heart rate ↓,	[70]

BMC: bone mineral content, BMD: body mass density, BMI: body mass index, BW: body weight, cd: cooling down, CHOL: cholesterol, CK: creatine kinase, DOMS: delayed onset muscle soreness, HDL: high-density lipoprotein, HOMA, HOMA-IR: homeostasis model assessment—insulin resistance, LDL: low-density lipoprotein, se: serum, tr: training, TG: triglyceride VSP-A: 'Vécu et Santé Perçue des Adolescents' (VSP-A) questionnaire is a French generic self-administered health-related quality of life (HRQL) questionnaire, wu: warming up, yr: year old. ↑, and ↓indicate an increase and a decrease, respectively. ^a^: number of total participants (participants in the eccentric group)

Bocalini et al. have found in elderly obese women that a 12-week exercise program could significantly improve body weight, BMI, body fat, and fat mass in the overweight and obese groups compared to the previous training values [63]. The changes in the trained obese group were so large that they were significant compared to the overweight and untrained obese groups [63]. Only the trained obese group has gained significant lean body mass [63]. However, in this study, the exercise program has not been completely eccentric. A single session of eccentric exercise, however, has failed to change body weight or BMI [62] (Table 2).

Longer periods, like four weeks of squatting, either eccentric or concentric training, significantly reduces waist circumference [69]. Twelve weeks of eccentric training on a dynamic resistance training machine significantly increases the lean body mass in overweight participants [70].

The eccentric training has also decreased the systolic blood pressure and heart rate in overweight or obese adults [68–70]. In elderly patients, descending stair walking has significantly decreased resting heart rate and systolic blood pressure [64].

In other studies, eccentric training has significantly decreased serum total cholesterol, LDL [68] and HDL, and triacylglycerol levels after one session of eccentric training [62]. Eccentric training significantly improves serum lipid parameters in elderly patients [64] (Table 2). However, in other cases, the advantageous effect of eccentric training on serum lipid parameters could not be seen [43, 70].

Eccentric training could normalize nonphysiological serum insulin and glucose levels as well as the HOMA index in adolescents [43]. Additionally, serum HbA1C level and AUC value of OGTT in the elderly [64] (Table 2) show that eccentric exercise may decrease the risks of developing diabetes. Patients in these studies have had no T2DM; therefore, they could not be included in our present study [68, 70].

Julian et al. have found that both eccentric and concentric cycling trainings are associated with positive changes in Health-Related Quality of Life and Health Perception [66]; thereby, they can modify mental health. In this study, eccentric exercise was more potent than concentric cycling training, which is probably not due to anthropometry, body composition, and functional changes [66].

Similarly to T2DM, the favorable effect of eccentric exercise on body mass, serum lipid panels, and circulatory parameters has also been observed in obesity.

## Eccentric Contraction-Induced Changes in Myokine Production

The more significant muscle damage of eccentric exercise is manifested by increased inflammatory cytokine release, such as interleukin-6 (IL-6), interleukin-1β, interleukin-8, interferon gamma-induced protein 10, and monocyte chemotactic protein 1 [71–73]. However, the identified skeletal muscle-derived cytokines may act locally and exert their effects in an autocrine or paracrine fashion. In addition to inflammatory cytokines, eccentric exercise triggers the production of skeletal muscle-derived IL-6 [74, 75]. IL-6 is a Janus-faced interleukin, which acts as both pro-inflammatory cytokine and anti-inflammatory myokine suppressing the production of the pro-inflammatory cytokine TNF-α [52, 76, 77]. Additionally, a growing body of evidence supports the concept that IL-6 might play a feasible role in exercise-related metabolic adaptations. It has been reported that IL-6 can induce GLUT4 translocation to the cell membrane in response to insulin-stimulated glucose disposal, and it can also improve glucose tolerance, possibly through the AMPK signaling pathway [78–80]. Several studies have reported eccentric exercise-mediated IL-6 release, which might serve as a possible link between eccentric training-related systemic adaptation and metabolic processes [81–83]. However, there is a controversy in the literature whether the magnitude of IL-6 response is more significant in eccentric exercise. A single bout of downhill running is characterized by profound muscle damage and enhanced IL-6 increment [81, 84]. Additionally, in the presence of low muscle glycogen, downhill running was able to provoke an unaltered IL-6 response [82], further supporting the role of IL-6 in glucose disposal and improved glucose tolerance. In another study, eccentric exercise has significantly increased serum IL-6 compared to the baseline levels, although this increment was markedly lower compared to an intensity-matched concentric training protocol [49].Whereas other studies have demonstrated that IL-6 levels are negatively correlated with eccentric exercise. Compared to the pre-exercise state, repeated bouts of eccentric knee extensions have failed to increase circulating IL-6 [85], while downhill running has even decreased IL-6 levels after the second bout of training [86]. Non-muscle-damaging downhill walking for 1 h has improved glucose tolerance in young, healthy males; however, it has failed to increase serum IL-6 compared to uphill walking [87].

Irisin is a cleaved fragment of fibronectin type III domain-containing protein 5 (FNDC5) and is secreted from muscles in response to exercise [88, 89]. An elevated level of irisin is associated with several beneficial effects on metabolic health, including glucose and fatty acid uptake in muscles, enhancement of the browning process of white adipose tissue, and enhanced thermogenesis via increasing the expression of uncoupling protein-1 (UCP-1) and induction of body mass loss [90–92]. Several studies have reported enhanced irisin response to various types of training protocols so far. However, only few studies have focused on the possible involvement of eccentric muscle contraction-induced irisin production and secretion. Downhill running has increased postexercise levels of serum irisin compared to level running training both in humans [84] and in rats [51]. Additionally, the intensity of the exercise program might trigger more significant irisin response [93]. In contrast, acute eccentric contraction of the knee extensor muscles has failed to modify serum irisin levels [94]. As a rule, even in the presence of metabolic syndrome, resistance exercise has induced the same irisin response as in healthy individuals [95], which further strengthens the assumption that the eccentric component of the applied training protocol exerts beneficial effects on metabolic health. When first identified, irisin has been proposed to be a PGC-1α (peroxisome proliferator-activated receptor gamma coactivator 1-alpha)-dependent myokine [88, 96]; therefore, increasing the expression of PGC-1α might be the initial signal for exercise-mediated irisin production and secretion. In rats, either a single bout or a six-week eccentric resistance exercise has elevated PGC-1α mRNA expression as well as serum levels of irisin, which has further been enhanced with β-hydroxy-β-methyl butyrate supplementation [97, 98]. Controversially, one acute bout of maximal single-leg eccentric knee extension exercise has failed to increase postexercise levels of PGC-1α mRNA in postmenopausal women [99].

IL-15 is believed to be a myokine that is implicated in the metabolic effects of eccentric exercise, and it is a highly expressed cytokine in skeletal muscles with essential roles in skeletal muscle growth and protein synthesis. Besides the anabolic effects, skeletal muscle-derived circulating IL-15 seems to reduce lipid accumulation in adipocytes; therefore, it is proposed to be a possible link in the exercise-mediated muscle-fat crosstalk [100, 101]. Plasma IL-15 has increased significantly in response to acute resistance exercise [102], high-intensity eccentric exercise [103], and eccentric cycling with preceding whole-body cryostimulation [104]. Aside from that, IL-15 may exhibit anti-inflammatory properties. A single bout of eccentric resistance exercise has markedly elevated plasma IL-15 levels in both athletes and non-athletes as well as attenuated serum TNF-α and C-reactive protein levels. This implies potential anti-inflammatory effects of IL-15 release during the exercise-induced inflammatory process [105]. Given that inflammation is recognized as an important factor in the development and progression of T2DM and obesity, the anti-inflammatory properties of eccentric exercise-driven IL-15 might serve as a promising modulator to attenuate the detrimental effects of these metabolic diseases.

## Discussion

**Fig. 2:**
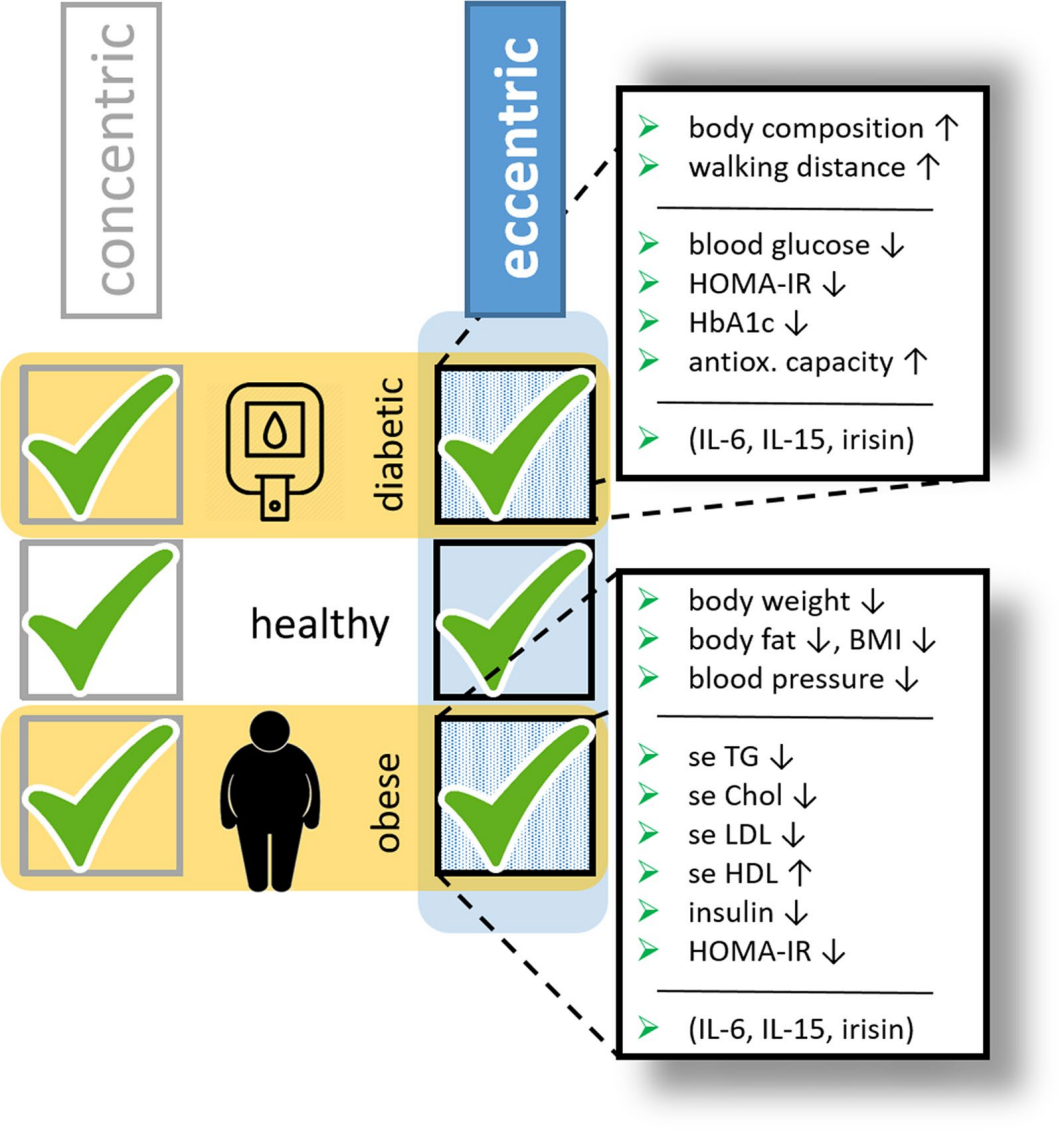
Effects of eccentric training on patients with type 2 diabetes or obesity. Eccentric contraction-based physical activity is an effective intervention to improve global health and provide additional and/or alternative protection against T2DM and obesity as shown by improved physiological and blood test parameters. Altered myokine production may play a role in the background mechanism. BMI: body mass index, HOMA-IR: homeostasis model assessment—insulin resistance, IL-6, IL-15: interleukin 6 and 15, respectively, se Chol: serum cholesterol, se HDL: serum high-density lipoprotein, se LDL: serum low-density lipoprotein, and se TG: serum triacylglycerol

Our working hypothesis in this work was that eccentric training, a unique form of skeletal muscle contraction can confer advantageous effects against various chronic metabolic diseases, such as T2DM and/or obesity and overweight in humans. This may be due to an altered contraction-induced myokine production, which may be responsible for the improved glucose homeostasis and weight loss. Therefore, we performed a PubMed search together with a Semantic Scholar search, focusing on human eccentric exercise studies with the primary outcomes related to blood glucose and lipid homeostasis, body composition, and functional tests. Altogether, we found only 10 papers related to T2DM or obesity. We could summarize that several forms of eccentric contraction-based intervention may confer some kind of effective protection against obesity and T2DM in humans (Tables 1 and 2, Fig. 2).

In this study, we investigated the effect of eccentric exercise on T2DM and obesity that have already developed. One can speculate that eccentric exercise may provide better protection against the development or early phase of T2DM and obesity when applied as a kind of preventive intervention. It is still to be confirmed that a longer duration of eccentric training (16-week duration was the longest) confers significant protection.

In this review, we found that eccentric training could release different myokines with systemic effects, e.g., various interleukins and irisin. These myokines together with others, e.g. meteorin-like protein, are regarded as exercise mediators in improving obesity-induced complications, such as insulin resistance, T2DM, and inflammation [106]. They are also accepted to increase insulin sensitivity, thereby improving glucose disposal and regulating glucose and lipid metabolism [107]. In a recent review, Marrano et al. have shown that irisin levels are lower in T2DM patients [108], which may confirm our findings about the moderate effect of eccentric training in T2DM and obesity. Sabaratnam et al. have, however, suggested that the potential beneficial metabolic effects of these myokines are not impaired in patients with T2DM [109]. Irisin administration, however, could augment insulin biosynthesis and promote the accrual of beta-cell functional mass, improve glycemic control, and promote weight loss in diabetic and/or obese animal models [108].

Eccentric exercise could modify the background mechanisms, e.g., increased serum sensitivity, antioxidant capacity, GLUT4 expression, improved lipid profile, decreased insulin resistance, and serum insulin content (Tables 1 and 2).

### Limitations of the eccentric exercise research field

This paper provides a recent update on the impact of eccentric exercise on whole-body metabolism in chronic metabolic diseases like prediabetes, T2DM, overweight, and obesity. However, it needs to be noted that this field has some severe limitations. First of all, there is no exact experimental definition of eccentric training since the intensity and types of eccentric training programs vary across the studies. An overview of the most commonly prescribed eccentric training methods for clinical studies is provided by Suchomel et al. [28, 110]. It is also nearly impossible to eliminate the potential impact of any other spontaneous physical activity (i.e., steps per day). According to our findings, another problem is the use of inappropriate terminology. The description of the training is sometimes confusing, particularly if the reader is not an expert in the field. Finally, in the elaborated studies, the small sample size of the experimental groups was also a limiting factor.

### Future perspectives

In recent years, exercise training and other forms of physical activity have become one of the main clinical interventions for the prevention and treatment of modern metabolic diseases (i.e., sport as medicine) [11]. Therefore, a better understanding of the mechanisms of eccentric training holds promise for the discovery of novel therapeutic targets and optimization of physical activity to become effective interventions to improve global health. Eccentric training standardization, however, is a critical issue for the planning of further clinical studies that specifically examine eccentric training in relation to different metabolic disease states [28, 110]. It is also crucial that eccentric training experiments should be conducted in animal models to discover, validate, and optimize novel therapeutics for their safe use in humans [111].

## Conclusion

Both in T2DM and obese patients, eccentric exercise seemed to improve (i) body composition (e.g., increased lean mass and decreased body weight, body fat, BMI, and fat mass); (ii) power (e.g., increased walking distance); and (iii) metabolism (e.g., increased fat oxidation, decreased carbohydrate oxidation, and serum triacylglycerol content).

In this review, we found that eccentric contraction-based physical activity might be an effective intervention to improve global health and provide additional and/or alternative protection against T2DM and obesity. However, further studies with large number of participants and standardized eccentric training protocols are needed to fully understand the protective effect of eccentric training against T2DM and obesity.

## Data Availability

Not Applicable.

## Abbreviations

BMI: Body mass index
FNDC5: Fibronectin type III domain-containing protein 5
GLUT: Glucose transporter
HOMA-IR: Homeostatic model assessment for insulin resistance
OGTT: Oral glucose tolerance test
PGC-1α: Peroxisome proliferator-activated receptor gamma coactivator 1-alpha
RENEW: Resistance exercise via negative eccentric work
T2DM: Type 2 diabetes mellitus
UCP-1: Uncoupling protein-1
